# Development of a virus neutralisation test to detect antibodies against Schmallenberg virus and serological results in suspect and infected herds

**DOI:** 10.1186/1751-0147-54-44

**Published:** 2012-08-07

**Authors:** Willie Loeffen, Sjaak Quak, Els de Boer-Luijtze, Marcel Hulst, Wim van der Poel, Ruth Bouwstra, Riks Maas

**Affiliations:** 1 Department of Virology, Central Veterinary Institute of Wageningen University and Research Centre (CVI-Lelystad), P.O. Box 65, 8200 AB, Lelystad, The Netherlands; 2 Livestock Research of Wageningen University and Research Centre, P.O. Box 65, 8200AB, Lelystad, The Netherlands

**Keywords:** Schmallenberg virus, Neutralisation test, Serology, Sensitivity, Specificity, Seroprevalence

## Abstract

**Background:**

At the end of 2011, a new orthobunyavirus, tentatively named Schmallenberg virus (SBV), was discovered in Germany. This virus has since been associated with clinical signs of decreased milk production, watery diarrhoea and fever in dairy cows, and subsequently also with congenital malformations in calves, lambs and goat kids. In affected countries, initial surveillance for the infection was based on examination of malformed progeny. These suspicions were followed up by real-time reverse transcription polymerase chain reaction (RT-PCR) on brain tissue. For epidemiological purposes, a serological assay was, however, needed.

**Results:**

A virus neutralisation test (VNT) was developed and optimized, and subsequently evaluated. This VNT has a specificity of >99% and the sensitivity is likely also very close to 100%. The assay is highly repeatable and reproducible. The final assay was used to test for antibodies in cows, ewes and does from herds known to be infected or suspected to be so. Targets for sampling in these herds were the mothers of malformed offspring. In herds with an RT-PCR confirmed SBV infection, more than 94% (190 out of 201) of the ewes and 99% (145 out of 146) of the cows were seropositive. In herds with suspicion of SBV infection based on birth of malformed offspring only (no or negative RT-PCR), more than 90% (231 out of 255) of the ewes and 95% (795 out of 834) of the cows were seropositive. In goats, on the other hand, only a low number of seropositives was found: overall 36.4%, being 16 out of 44 goats tested.

**Conclusions:**

Given the characteristics of this VNT, it can be used at a relative high throughput for testing of animals for export, surveillance, screening and research purposes, but can also be used as a confirmation test for commercially available enzyme-linked immunosorbent assays (ELISA’s) and for (relative) quantification of antibodies.

Suspicions of SBV infections that were confirmed by RT-PCR were almost always confirmed by serology in cows. Due to individual registration and identification of cows and calves, affected offspring could almost always be traced back to the mother. Ewes on the other hand were not always the mothers of affected lambs, but were in many cases herd mates with unaffected lambs. This indicated a high within-herd seroprevalence of antibodies against SBV.

## Background

On the 18^th^ of November 2011, the finding of a new orthobunyavirus was reported by the Friedrich Loeffler Institute (FLI) in Germany [1]. This virus is closely related to Shamondavirus, which belongs to the Simbu serogroup of the genus *Orthobunyavirus*, family *Bunyaviridae*[2]. The virus, provisionally called Schmallenberg virus (SBV), has since been associated with clinical signs of decreased milk production, watery diarrhoea and fever that had occurred in the months of August and September in dairy cows in the Netherlands [3] and Germany [1]. In Germany, twelve samples from six affected herds that were tested with a real-time reverse transcription polymerase chain reaction test (RT-PCR) were positive for viral RNA of SBV [1]. In the Netherlands, using the same RT-PCR, viral RNA of SBV was detected in 36% of stored blood samples from herds that had reported clinical signs earlier. In contrast, none of the samples from herds without clinical signs in the Netherlands were RT-PCR positive for SBV [3].

Subsequently, SBV infections were also associated with congenital arthrogryposis and hydranencephaly syndrome in newborn lambs [4]. A first malformed goat kid was reported PCR-positive for SBV on 3 January 2012 in the Netherlands [5] and the first malformed calf was reported PCR-positive for SBV on 7 January 2012 in Germany [6].

To determine the seroprevalence and carry out epidemiological studies, the immediate need arose for a reliable and robust serological assay. To address this need, a virus neutralisation test (VNT) was developed, as this also allows for a semiquantitative detection of antibodies against the virus. Furthermore, a protocol was established that allows for a relatively high throughput. Even though in April 2012 a first commercial enzyme-linked immunosorbent assay (ELISA) became available, a VNT remains a possible alternative as a screening test, but is also useful as a confirmation test or a semiquantitative test to be used in experimental infections, when amounts of antibodies need to be determined. The development of the VNT is described, evaluating the diagnostic specificity and sensitivity, repeatability and reproducibility, and robustness, and use of the test on RT-PCR positive herds and herds suspected to be infected, i.e. occurrence of malformed progeny, with negative RT-PCR results or RT-PCR not performed.

## Methods

### Medium

The medium used was DMEM + Glutamax (Gibco, Bleiswijk, the Netherlands) with 3% foetal calf serum (FCS), penicillin (100 units/ml), and streptomycin (100 μg/ml). FCS was inactivated for 30 min at 56°C. This medium was used in all cell cultures and to dilute serum samples and virus stock.

### Cell culture

Vero cells were used to grow the virus and also in the VNT. They were grown in 600 ml (150 cm^2^) cell culture flasks (Becton Dickinson, Breda, the Netherlands). Depending on the need for cells and availability of cells, flasks were seeded with 6 to 10 million cells, with 50 ml of medium, and incubated at 37°C, in an atmosphere of 5% CO_2_. Cells for the VNT were counted with a cell counter (Beckman Coulter, Woerden, the Netherlands) and diluted to a concentration of 2x10^5^ cells/ml.

### Virus

The virus for the VNT was isolated from the brain of a new-born lamb with malformations that had tested positive in the SBV RT-PCR. A virus stock containing approximately 10^5^ median tissue culture infective dose (TCID_50_) per ml (3^rd^ passage) was grown initially on Vero cells and used to develop and optimize the VNT. A second, larger batch of virus, with a titre of approximately 10^6.7^ TCID_50_/ml (4^th^ passage) was grown also on Vero cells and used for the initial validation of the final test protocol and subsequent routine diagnostic tests. Titres were determined by amido black staining, similar to back titration of the virus in the VNT (see paragraph “Back titration of virus”).

### Development of the VNT

Several variables were changed and tested during the development of the assay to determine the optimal conditions, but also the robustness of the VNT:

Number of cells per well: 5000, 10,000, 20,000, and 50,000.

Age of cells (time to harvesting after incubation): 3 to 16 days.

Percentage of FCS in medium: 1%, 3%, and 5%.

Neutralisation time: 1, 2, and 4 hours.

Incubation period: 3, 4, and 5 days.

Amount of virus: 100 and 500 TCID_50_.

Two different batches of FCS.

Microscopic reading of the plates without staining (cytopathic effect [CPE]) *vs*. macroscopic reading of the plates after amido black staining.

### Final VNT protocol

The VNT was carried out in flat bottomed 96 well micro titre plates. In each well of the first column, 75 μl of medium was pipetted, in all other wells 50 μl of medium. Serum samples were inactivated for 30 min at 56°C and 25 μl was subsequently added to a well in the first column to obtain a starting dilution of 1:4. From this well, two-fold dilutions were made by pipetting 50 μl of each well in the next, until the last column. From the final column, 50 μl was discarded, so that each well now contained 50 μl of the diluted sample.

Subsequently, virus (500 TCID_50_ per well) was added to each well, also in a volume of 50 μl. Serum and virus were pre-incubated at 37°C for 1 to 2 hours to allow neutralisation of the virus. Thereafter, 20,000 cells per well were added in a volume of 100 μl. Plates were then incubated for 5 days at 37°C and under 5% CO_2_.

After 5 days, the plates were emptied, washed once with 0.15 M NaCl and then stained with amido black (0.1% amido black solution (w/w) with 5.4% acetic acid, 0.7% sodium acetate and 10% glycerol) for 30 min. Washing fluids were caught in a container with disinfectant, to inactivate the virus. Subsequently, plates were rinsed with tap water and read macroscopically. Wells with 25-100% staining were considered to have no or limited CPE only. Wells with less than 25% staining were considered to have extensive or full CPE. In each dilution series the last well with no or limited CPE (25-100% staining) was identified and the sample was assigned a titre that was the reciprocal of the dilution in that well.

### Control samples

Control samples included serum samples from 2 seronegative sheep, 4 seropositive sheep and 2 seropositive dairy cows. The positive samples were obtained from sheep and dairy cows from herds in which congenital malformations were seen and confirmed by RT-PCR. The samples had tested consistently positive in subsequent prototypes of the VNT. These control samples were included in each run of the test and were also used to optimize the assay.

### Back titration of virus

Virus used in each run of the test was back titrated in 4 tenfold dilutions. If a working stock of 100 TCID_50_/well was used in the test, 10^0^, 10^-1^, 10^-2^, and 10^-3^ dilutions of that working stock were used in the back titration. If a working stock of 500 TCID_50_/well was used in the test, 10^-1^, 10^-2^, 10^-3^ and 10^-4^ dilutions of that working stock were used in the back titration. Each dilution was tested in 12 or 24 wells during the development phase and routine diagnostic phase respectively. The virus titre was determined with the Reed-Münch method [7].

### Diagnostic specificity and sensitivity

To determine the optimal cut-off of the assay, with a high diagnostic specificity and sensitivity, 348 blood samples from non-infected animals were tested in the final version of the assay. These samples originated from the Netherlands from the period of 2000–2010, supposedly before the introduction of SBV. From sheep 156 samples were available and 192 from cattle. They were used to set the cut-off value in the assay and estimate the specificity. Subsequently, 366 field samples were tested that originated from herds in which congenital malformations were observed to get a first indication of the sensitivity of the test.

### Repeatability and reproducibility

To test for repeatability of the assay, 646 samples from suspect and infected herds were tested in duplicate, in 8 dilutions. The number of mismatches between duplicates was tabulated to get a first indication of the repeatability. For reproducibility, the results from the control panel of 4 runs were used. For each sample the standard deviation of the log-transformed titres was calculated. The standard deviations of all 8 samples were averaged, to get a first indication of the reproducibility.

### Field study

From the end of February 2012 both an RT-PCR and the VNT were used as routine diagnostic tests to confirm or rule out SBV infections in case of congenital malformation. The samples used in this study were collected in the field from the end of December 2011 until the 14^th^ of May 2012. Common procedure was that malformed calves, lambs and goat kids would be submitted for a post-mortem. If an infection with SBV was suspected, based on arthrogryposis and hydranencephaly, a brain sample was tested by RT-PCR for SBV, while the competent authorities would trace the mother of the affected offspring to take a blood sample for serological testing. However, tracing of the mother was not always possible, especially in sheep due to lack of individual registration of mothers and lambs at that age. Therefore, in many cases a ewe that was not the mother of the malformed lamb would be sampled from the herd instead. Until mid-May, a total number of 1480 blood samples were tested in the VNT, originating from 1205 different herds (30 goat, 259 sheep and 916 cattle herds). These herds were divided in two categories:

Suspect herds, being herds with occurrence of malformed progeny, but either no or only negative RT-PCR results.

Infected herds, being herds with occurrence of malformed progeny, and a positive RT-PCR result.

All samples were tested in the final VNT-protocol that was evaluated as described above.

## Results

### Development of the assay and robustness

Several variables were tested and evaluated while developing and optimizing the assay. Percentage of FCS (from 1-5%), neutralisation time (1–4 hours), age of cells (3 to 16 days), and different batches of FCS gave the same result and were not crucial within the tested range. The percentage of FCS was therefore fixed at 3%, with a neutralisation time of 1–2 hours for the final protocol. The test is, however, robust for these variables, within given ranges.

The number of cells, on the other hand, was crucial. A total of 20,000 cells per well gave confluent monolayers and full staining of non-affected cells, while 5,000 or 10,000 cells per well gave rather thin and sometimes incomplete monolayers. Especially for easiness of reading, 20,000 cells per well was included in the final protocol, as 50,000 cells per well gave no further improvement.

Microscopic reading of the plates turned out to be laborious and quite difficult to standardize between observers who were reading the test. Given the need for a relatively high throughput VNT, this was abandoned in favour of amido black staining and macroscopic reading of the plates. Even though CPE was defined as 75% of the monolayer gone, which seems a somewhat subjective criterion, this was very easy to standardize between observers and rarely resulted in discrepancies.

**Figure 1:**
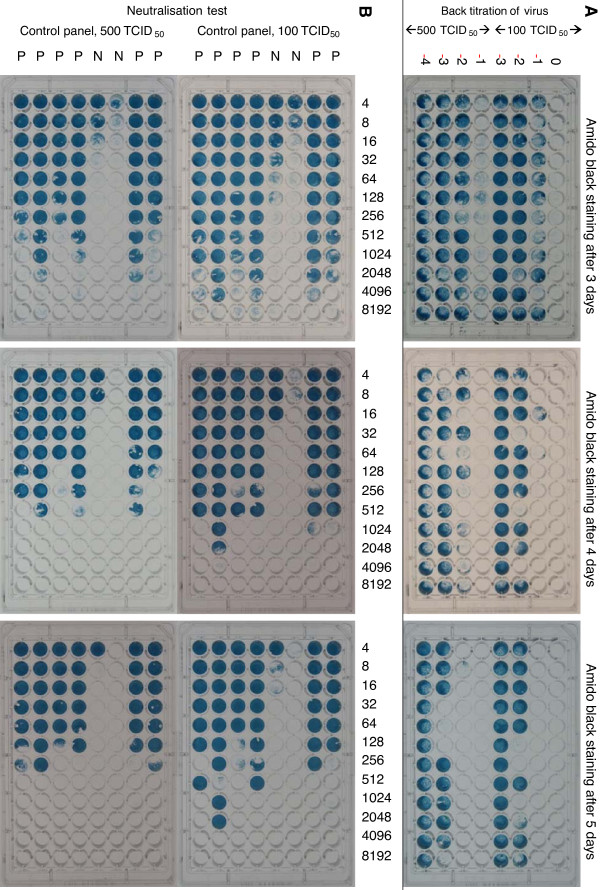
**Amido black staining of control plates, for comparison of 100 vs. 500 TCID**_**50**_**and 3, 4 and 5 days of incubation.A**: Back titrations of both virus concentrations, shown in the upper 3 plates, were carried out on one plate per incubation period, in 4 tenfold dilutions starting from 10^0^ (100 TCID_50_, upper 4 rows) or 10^-1^ (500 TCID_50_, lower 4 rows). **B**: Results of the control panel of 6 positive (P) and 2 negative (N) samples, are shown in the middle row of 3 plates (100 TCID_50_) and the lower row of 3 plates (500 TCID_50_). Serum dilutions are shown above the figures. Results of back titration and average titres are given in Table 1.

For the amido black staining, crucial differences were noticed when using different virus concentrations and incubation periods. These results are shown in Table 1 and Figure 1, where test plates were prepared in triplicate, of which one plate was stained and read after 3, 4 and 5 days respectively. In Table 1 it is shown that back titration of the virus yields higher titres when plates are read microscopically and that higher titres are observed after longer incubation periods. Serum titres are higher when using 100 TCID_50_ instead of 500 TCID_50_ (as can be expected), and serum titres are decreasing after longer incubation periods, as more CPE develops. In Figure 1 it is clearly shown that incubation for a longer period results in plates that are easier to read. Especially after 5 days, and with 500 TCID_50_, there is a good contrast between wells with and without CPE. This is even more so the case for the back titration of the virus. An incubation period of 5 days and 500 TCID_50_ were therefore included in the final protocol.

**Table 1:** **Back titrations (in TCID**_**50**_**/well) and average titres of positive (n = 6) and negative (n = 2) samples, depending on intended TCID**_**50**_**per well (100 vs. 500) and days of incubation of the VNT (3, 4, or 5 days)**

		**100 TCID_50_**			**500 TCID_50_**		
		**3 days**	**4 days**	**5 days**	**3 days**	**4 days**	**5 days**
Virus titres							
Back titration	Microsc	59	154	202	314	720	1389
	AmidoB	7	41	56	35	187	562
Antibody titres							
Positive samples	Microsc	211	215	181	78	57	51
	AmidoB	2048	813	287	575	228	181
Negative samples	Microsc	3	4	3	3	2	3
	AmidoB	16	6	3	6	4	3

Plates were read in two ways: microscopic (Microsc), where CPE was defined as even a single plaque of CPE, and with amido black staining (AmidoB), where CPE was defined as 75 % or more of the monolayer gone (is <25% staining).

### Diagnostic specificity of the VNT

**Figure 2:**
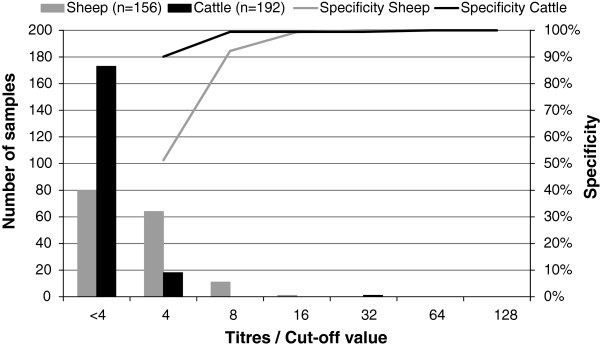
**Distribution of titres in non-infected sheep (n = 156) and cattle (n = 192) (bars, left axis).** Serum samples were tested in two fold dilutions from 1:4 until 1:128. Specificity of the VNT in sheep and cattle serum samples, depending on cut-off value (a cut-off value of 4 meaning that all titres of 4 and above are considered positive, etc.) for sheep and cattle is shown with the lines (right axis).

In serum samples from non-infected sheep, a higher background was seen than in sera from non-infected cattle (Figure 2). Based on testing 156 sheep and 192 cows, the cut-off values were set at a titre of 16 for sheep and 8 for cattle. Given this cut-off value, a specificity of 99.4% (95% CI: 96.6-99.9%) in sheep and 99.5% (95% CI: 97.1-99.9%) in cattle was estimated (Figure 2). No negative samples from goats were available, and the cut-off value was set to 16, similar to sheep.

### Diagnostic sensitivity of the VNT

**Figure 3:**
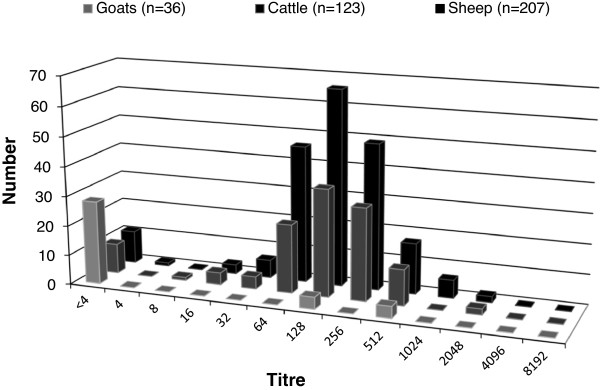
Distribution of titres in goats, sheep and cattle in a total of 366 samples from herds with congenital malformations.

Due to the lack of well-defined serum samples, no real estimate of the diagnostic sensitivity was initially possible. From the 366 field samples that were tested during the evaluation of the final test protocol, 92% of the cattle and 94% of the sheep scored positive in the VNT. In sheep no titres of 8 and in cattle no titres of 4 were observed, thus clearly separating the peak of the positive samples from the peak of the negative samples (Figure 3). This supports the cut-off value of 16 and 8 respectively for sheep and cattle, as determined already by testing serum samples from non-infected animals. Assuming the worst case scenario, that all were seropositive in reality, sensitivity of the assay would therefore be at least 92% and 94% in cattle and sheep respectively. However, the clear separation of the negative and positive peak suggests that the sensitivity for both species is close to 100%. In goats only 8 samples were positive, out of 36 samples tested.

### Repeatability and reproducibility

From 646 samples tested in duplicate, 398 (61.6%) were identical. In 235 samples (36.4%) there was one dilution step difference between the duplicates and in 11 samples (1.7%) the difference was two dilution steps. In two samples (0.3%) the difference was 5 and 6 dilution steps respectively, and these were the only 2 samples in which the qualitative result (positive/negative) would be different if the result was to be based on either of the duplicates. Based on qualitative results, the repeatability was therefore 99.7%.

The average standard deviation of the control samples in 4 different test runs was 0.52 log_2_, which is slightly more than half a dilution step. Titres were therefore reproducible on average with a range of plus or minus half a dilution step.

### Field study

In cattle, 135 out of 916 herds with occurrence of malformations (14.7%) were confirmed infected with SBV by RT-PCR. In infected herds, 99.3% of the cows tested positive in the VNT, while 95.3% were seropositive in suspect herds (Table 2).

**Table 2:** Result of a serological field study in cattle, sheep and goat herds, carried out with the VNT

		**Herds**				**Blood samples**	
		**Tested**	**PCR+**	**PCR-**	**No PCR test**	**Tested**	**Positive (%)**
Cattle	Total	916	135	751	30	980	940 (95.9 %)
	Suspect	781	-	751	30	834	795 (95.3 %)
	Infected	135	135	-	-	146	145 (99.3 %)
Sheep	Total	259	79	168	12	456	421 (92.3 %)
	Suspect	180	-	168	12	255	231 (90.6 %)
	Infected	79	79	-	-	201	190 (94.5 %)
Goats	Total	30	3	22	5	44	16 (36.4 %)
	Suspect	27	-	22	5	35	14 (40.0 %)
	Infected	3	3	-	-	3	2 (66.7 %)

Suspect herds being herds with occurrence of malformed offspring, but either negative SBV RT-PCR results on a herd level, or no RT-PCR results. Infected herds being herds with occurrence of malformed offspring and a positive SBV RT-PCR result.

In sheep, 79 out of 259 herds with occurrence of malformations (30.5%) were confirmed infected with SBV by RT-PCR. In infected herds, 94.5% of the ewes tested positive in the VNT, while 90.6% were seropositive in suspect herds.

In goats, fewer cases of malformations were reported and investigated, and in 3 out of 30 herds (10%) SBV was confirmed by RT-PCR. Two out of three does tested in these infected herds were positive in the VNT. In suspect herds, 40% of the does tested positive in the VNT.

## Discussion

A very robust VNT was developed with a very high specificity and sensitivity, both close to 100%, in sheep and cattle. The assay was shown to be highly repeatable and reproducible. The VNT is easy to perform and can be read macroscopically as a result of the amido black staining. With the current, optimized protocol, this staining results most of the times in a very sharp transition between wells with and without CPE. This makes it easy and very fast to read the plates, with a high reliability and reproducibility. A serum control for each individual sample was not included during our evaluation of the test. No evidence for a toxic effect of serum on the cells was ever noticed, but to avoid false negative results, routine use of a serum control could be considered.

In infected herds, i.e. confirmed by RT-PCR, more than 94% of the sheep and 99% of the cows tested seropositive. The ewes and cows tested were sampled because of congenitally malformed offspring. In lambs, approximately 30% of these malformations were confirmed to be positive for SBV by RT-PCR on brain tissue. However, in many cases it was not possible to trace the confirmed SBV-positive lambs back to the exact mother, so the serum blood sample was sometimes obtained from other ewes in the herd. Nevertheless, more than 94% of these ewes tested seropositive, suggesting that at least within these sheep herds with congenital malformations, the infection is widespread with a high within-herd seroprevalence.

Calves with malformations, on the other hand, could usually be traced back to the mother and from these cows serum blood samples were collected for serology. More than 99.3% of these mothers were seropositive. These cattle sera originated from cows that gave birth to malformed calves that were positive in the RT-PCR for SBV and are currently the most well-defined serum samples from previously infected animals. The sensitivity of the VNT can be estimated most reliably from these samples and is therefore >99%, which is in line with the estimation during the initial evaluation of the test, based on the distribution of titres in a smaller set of field samples.

In suspect herds, SBV was not confirmed by RT-PCR and in some cases RT-PCR was not carried out at all. It is likely that the virus, after infecting the foetus and causing the malformations, was cleared during the last part of the gestation period, at least from the brain, which was the most common sampling site. The fact that calves were more often RT-PCR negative than lambs can then be explained by the longer gestation period in cows and supports this explanation. The fact that seroprevalences in suspect herds were slightly lower than in infected herds, suggests that also malformed offspring with defects unrelated to a SBV infection were submitted for testing. Given the high alertness for congenital malformations, this can be expected to happen. In any case it shows that serological assays are needed to reliably detect infections.

High seroprevalences, both on a regional level, but also within herds, with and without obvious clinical signs, are not uncommon for viruses related to SBV. In Australia, seroprevalence studies into the related Akabane virus (AKAV), revealed within-herd seroprevalences of 77% in 1964 [8], up to 89% in 1971 [9] and 99% in 1988 in the New South Wales area [10]. Furthermore, in Japan, 74% of apparently healthy cattle cohabitated with cows showing clinical signs from an AKAV infection were seropositive also [11]. Finally, a very high seroprevalence is reported in the Netherlands, both within the Netherlands as a whole as in a few individual herds [12].

In goats the number of seropositive animals is far lower than in sheep and cattle, although less samples from goats were tested and the estimated seroprevalence is therefore less precise. Most of the malformations in goats therefore likely had another cause, as no infection with SBV could be proven in most of the mothers of the affected kids, neither by RT-PCR, nor by serology. Probably these affected kids were submitted and tested for SBV due to the ongoing outbreak and the high alertness for congenital malformations. These results may reflect a lower seroprevalence in goats in general. Given that goats, in contrast to cattle and sheep, are often housed indoors, and the SBV is supposedly transmitted by *Culicoides* vectors, like other viruses of the Simbu serogroup [2,13,14], this lower seroprevalence would not be surprising.

Many countries outside the EU have closed their borders for cattle and sheep from SBV infected countries. Importing countries may require testing of animals originating from infected countries, which could include RT-PCR and/or serological testing. Although ELISA’s are being developed, and one became recently available on the market, the VNT that was developed, is a possible alternative. It is easy to carry out, and even though the incubation period is quite long with 5 days, hands-on time is relatively short. This means that the VNT is relatively cheap, and the total test capacity for an average laboratory to carry out the VNT could be quite high. Depending on the number of dilutions to be tested, whether samples will be tested in duplicate or not, and based on existing experience and logistics within a laboratory, thousands of samples could be tested per week. Costs of the VNT are mainly related to labour, while costs of an ELISA are for a large part expenditures on materials. Furthermore, the VNT could be used as a confirmation test and probably even a gold standard if further evaluated and validated. For (semi)quantitative studies, either in animal experiments or field studies, a VNT also has its advantages.

## Conclusion

A very robust VNT was developed with a very high specificity and sensitivity, both close to 100%, in sheep and cattle. The assay was shown to be highly repeatable and reproducible. The VNT is easy to perform and can be read macroscopically as a result of the amido black staining. As such it can be used as a confirmation test for commercially available enzyme-linked immunosorbent assays (ELISA’s) and for (relative) quantification of antibodies, but also at a relative high throughput for testing of animals for export, surveillance, screening and research purposes.

## Competing interests

The authors declare that they have no competing interests.

## Authors’ contributions

WL, SQ, EB and RM designed the study. SQ and EB carried out all the laboratory work. MH isolated and propagated the virus. WL compiled the results, analysed the data and wrote the manuscript. All authors were involved in the interpretation of results and have given helpful advice in writing the paper. All authors read and approved the final manuscript.
